# MDA5 RNA-sensing pathway activation by *Mycobacterium tuberculosis* promotes innate immune subversion and pathogen survival

**DOI:** 10.1172/jci.insight.166242

**Published:** 2023-10-23

**Authors:** C. Korin Bullen, Alok K. Singh, Stefanie Krug, Shichun Lun, Preeti Thakur, Geetha Srikrishna, William R. Bishai

**Affiliations:** Center for Tuberculosis Research, Department of Medicine, Johns Hopkins School of Medicine, Baltimore, Maryland, USA.

**Keywords:** Immunology, Infectious disease, Innate immunity, Tuberculosis

## Abstract

Host cytosolic sensing of *Mycobacterium tuberculosis* (*M*. *tuberculosis*) RNA by the RIG-I–like receptor (RLR) family perturbs innate immune control within macrophages; however, a distinct role of MDA5, a member of the RLR family, in *M*. *tuberculosis* pathogenesis has yet to be fully elucidated. To further define the role of MDA5 in *M*. *tuberculosis* pathogenesis, we evaluated *M*. *tuberculosis* intracellular growth and innate immune responses in WT and *Mda5^–/–^* macrophages. Transfection of *M*. *tuberculosis* RNA strongly induced proinflammatory cytokine production in WT macrophages, which was abrogated in *Mda5^–/–^* macrophages. *M*. *tuberculosis* infection in macrophages induced MDA5 protein expression, accompanied by an increase in MDA5 activation as assessed by multimer formation. IFN-γ–primed *Mda5^–/–^* macrophages effectively contained intracellular *M*. *tuberculosis* proliferation to a markedly greater degree than WT macrophages. Further comparisons of WT versus *Mda5^–/–^* macrophages revealed that during *M*. *tuberculosis* infection MDA5 contributed to IL-1β production and inflammasome activation and that loss of MDA5 led to a substantial increase in autophagy. In the mouse TB model, loss of MDA5 conferred host survival benefits with a concomitant reduction in *M*. *tuberculosis* bacillary burden. These data reveal that loss of MDA5 is host protective during *M*. *tuberculosis* infection in vitro and in vivo, suggesting that *M*. *tuberculosis* exploits MDA5 to subvert immune containment.

## Introduction

Tuberculosis (TB) remains a serious health challenge, causing an estimated 1.5 million deaths worldwide in 2021 alone (1). *Mycobacterium tuberculosis* (*M*. *tuberculosis*) is exquisitely human adapted, with an exclusive niche for intracellular survival in macrophages through immune subversion mechanisms that remain incompletely understood. Initial engulfment of *M*. *tuberculosis* by lung macrophages activates the cytosolic surveillance pathway composed of germline-encoded pattern recognition receptors (PRRs), leading to increased type I interferon (IFN) and proinflammatory cytokine production, inflammasome activation, and autophagy (2–4). Studies from our laboratory and others showed that *M*. *tuberculosis* DNA and mycobacterially derived cyclic dinucleotides activate cytosolic DNA-sensing pathways (5–8), driving the expression of type I IFNs.

While the role of cytosolic viral RNA in innate immune sensing has been extensively studied, the contribution of bacterial RNA to disease pathogenesis is less well understood (9). The best-characterized RIG-I–like receptor (RLR) family members, RIG-I and melanoma differentiation factor 5 (MDA5), contain a central ATPase-containing DExD/H-box helicase domain and a C-terminal repressor domain, both of which are involved in RNA binding (10, 11). Upon activation through RNA engagement, 2 tandem caspase activation and recruitment domains (CARDs) interact with the adaptor mitochondrial antiviral signaling protein (MAVS) to mediate induction of NF-κB and IFN regulatory factors (IRFs) and the subsequent expression of IFN-stimulated genes (ISGs) (12–14). Despite their structural similarity, RLRs detect distinct RNA species that are often pathogen specific but not necessarily mutually exclusive (11, 15).

Growing evidence supports a nonredundant role for RIG-I in the type I IFN response to *M*. *tuberculosis* infection (16–18), through its binding to specific *M*. *tuberculosis* RNA transcripts that gain access to macrophage cytosol using the mycobacterial ESX-1 secretion system (16). We recently showed that *M*. *tuberculosis* infection in human macrophages increases RIG-I activation and phosphorylation at the S855 residue (18). Mice lacking MAVS protein, the RIG-I downstream signaling adaptor, show attenuated *M*. *tuberculosis* growth and prolonged survival compared with WT mice (16), similar to that observed in IRF3^−/−^ and Ifnar1^−/−^ mice (19, 20). RIG-I signaling has been shown to facilitate either *M*. *tuberculosis* survival in IFN-γ–primed mouse J774.1 macrophages or host control in human THP-1 monocytes (17, 18). While this difference might be species dependent, considering that type I IFNs appear to inhibit host control of *M*. *tuberculosis* infection (18), it is likely that the activation state of macrophages influences the effect of RLR-dependent innate immune sensing on intracellular *M*. *tuberculosis* survival (21).

Several lines of evidence also support a role for MDA5 during *M*. *tuberculosis* infection. Our recent phosphoproteomic screen during *M*. *tuberculosis* infection in human macrophages revealed enrichment of MDA5 phosphorylation at S301 (18), similar to concomitant findings showing MDA5 phosphorylation and ubiquitylation status changes in mouse primary macrophages during *M*. *tuberculosis* infection (22). *MDA5* transcript levels are significantly elevated in whole blood and lymph tissues from patients with active TB relative to healthy controls (23, 24). While bacteria-specific phenotypic outcomes of *Mda5* gene deficiency have been described (17, 25, 26), the molecular mechanisms regulating MDA5 signaling and the downstream consequences during obligate intracellular bacterial infection remain largely undefined. Here we show that the activation of MDA5 signaling facilitates *M*. *tuberculosis* survival in macrophages and that *Mda5^–/–^* mice display increased resistance to *M*. *tuberculosis* infection compared with WT mice. Our findings support the conclusion that *M*. *tuberculosis* co-opts MDA5 signaling to promote its own survival in the host through a culmination of proinflammatory cytokine production, inflammasome activation, and inhibition of autophagic pathways.

## Results

### MDA5 senses M. tuberculosis RNA.

**Figure 1:**
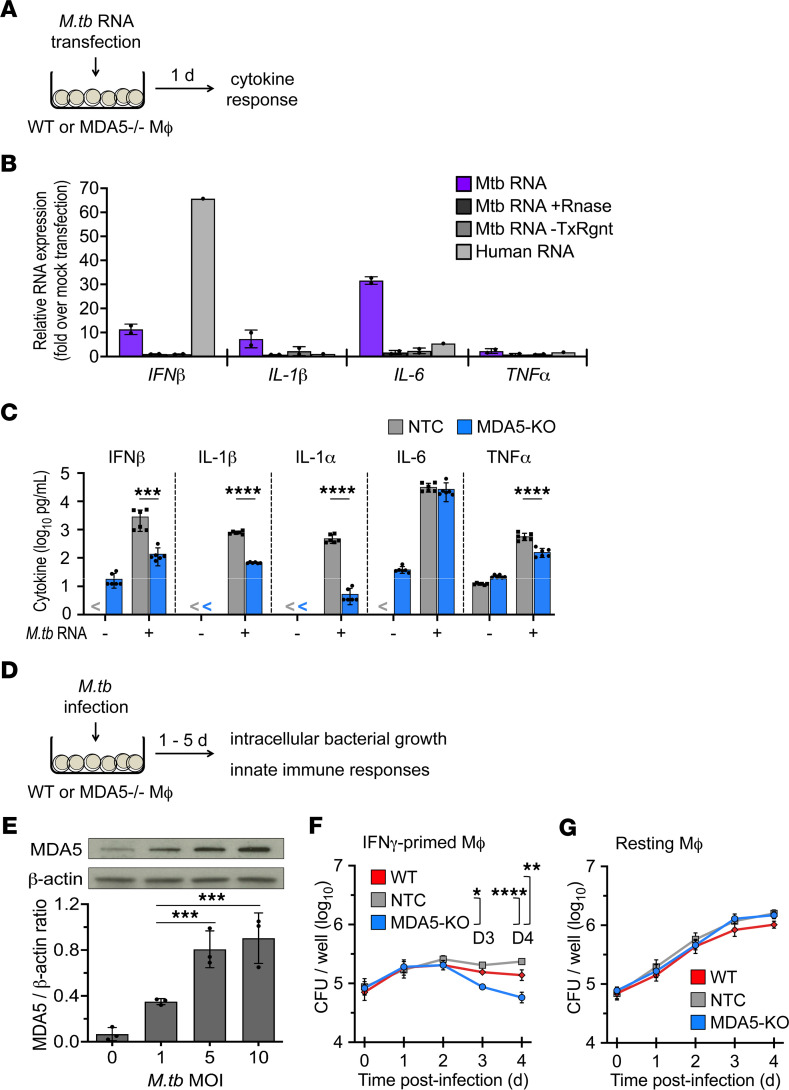
Cytosolic RNA sensor MDA5 modulates host cytokine response to *M*. *tuberculosis–*derived RNA and promotes *M*. *tuberculosis* intracellular growth in macrophages. (**A**) Schematic diagram of experimental design to identify host factors involved in cytosolic surveillance of *M*. *tuberculosis* RNA in macrophages. (**B**) Resting primary human monocyte-derived macrophages were transfected for 24 hours with total *M*. *tuberculosis* RNA, total *M*. *tuberculosis* RNA treated with RNaseV (+RNase), mock transfection of total *M*. *tuberculosis* RNA (–TxRngt), or total human RNA. IFN-β, IL-1β, IL-6, and TNF-α RNA levels were assessed by reverse transcriptase qPCR. RNA levels were normalized to PolR2A. Data are presented as fold-change relative to no-transfection control (mean ± SD, *n* = 2 biological replicates, 2 independent experiments). (**C**) CRISPR/Cas9–mediated *Mda5*-knockout (*Mda5*-KO) or CRISPR/Cas9 nontarget control (NTC) J774.1 cells were IFN-γ–primed and transfected with total *M*. *tuberculosis* RNA for 24 hours. IFN-β, IL-1β, IL-6, and TNF-α levels in the culture medium were quantified by multiplex immunoassay (Luminex) (mean ± SD, *n* = 6 biological replicates from 3 independent experiments; “<” indicates below limit of quantification). ***P < 0.001, ****P < 0.0001 by 2-way ANOVA with Tukey’s posttest. (**D**) Schematic diagram of experimental design to evaluate the role of *Mda5* in innate immune responses to *M*. *tuberculosis* infection. (**E**) MDA5 and β-actin protein levels in WT J774.1 cells at 24 hours after infection with *M*. *tuberculosis* at the indicated MOI were detected by immunoblot analysis and quantified by densitometry (mean ± SD, *n* = 3 biological replicates, 3 independent experiments). ***P < 0.001 by 1-way ANOVA with Tukey’s posttest. (**F** and **G**) Growth kinetics of *M*. *tuberculosis* (MOI of 1:5) in IFN-γ–primed (**F**) and resting (**G**) WT, NTC, and *Mda5*-KO J774.1 cells. Data are mean CFU ± SEM for each time point (*n* = 4 biological replicates, 2 independent experiments). *P < 0.05, **P < 0.01, ****P < 0.0001 by 2-way ANOVA with Tukey’s posttest.

To investigate whether *M*. *tuberculosis* RNA triggers MDA5 signaling, we transfected primary human monocyte-derived macrophages (hMDMs) with total RNA purified from the *M*. *tuberculosis* strain H37Rv, then measured mRNA levels of the proinflammatory cytokines IFN-β, IL-1β, TNF-α, and IL-6 by reverse transcriptase quantitative PCR (qPCR) 24 hours after transfection (Figure 1A). Transfected *M*. *tuberculosis* RNA, but not human RNA, induced IL-1β and IL-6 expression (Figure 1B). Similar results were observed in J774.1 mouse macrophages (Supplemental Figure 1A; supplemental material available online with this article; https://doi.org/10.1172/jci.insight.166242DS1). This response was completely abrogated in cells transfected with *M*. *tuberculosis* RNA treated with RNaseV, confirming that the observed cytokine response was an RNA-specific response and not a potential microbial contaminant (Figure 1B). No change was observed in macrophages exposed to *M*. *tuberculosis* RNA in the absence of transfection reagent, suggesting that intracellular localization is necessary for this response (Figure 1B). Further, cells transfected with human RNA purified from THP-1 macrophages showed no proinflammatory response (Figure 1B and Supplemental Figure 1A), indicating that the differential induction of proinflammatory cytokine expression was not a generalized cellular response to exogenous RNA. The abundance of RNA relative to any trace genomic DNA contaminants in the RNA extracts was confirmed by qPCR for 5S and 16S rRNA transcripts with and without no–reverse transcriptase (NRT) controls (10^5^-fold increase over NRT controls) (Supplemental Figure 1B). These results suggested that cytosolic *M*. *tuberculosis* RNA induced PRR-mediated immune responses in macrophages.

To determine the role of MDA5 in host sensing of *M*. *tuberculosis* RNA, we generated a clonal *Mda5*-KO J774.1 macrophage cell line using CRISPR/Cas9 nuclease RNA-guided genome editing, verified by DNA sequencing and immunoblot analysis (Supplemental Figure 2, A and B). IFN-γ–primed *Mda5*-KO and CRISPR/Cas9 nontarget control (NTC) macrophages were transfected with *M*. *tuberculosis* RNA, and levels of IFN-β and other proinflammatory cytokines were quantified by multiplex immunoassays at 24 hours after transfection. We observed a significant reduction in IFN-β, IL-1α, IL-1β, and TNF-α secretion by *Mda5*-KO macrophages compared with NTC cells in response to *M*. *tuberculosis* RNA transfection (Figure 1C), indicating a role for MDA5 in sensing immunogenic *M*. *tuberculosis* RNA species. The response from unprimed, resting NTC and *Mda5*-KO macrophages was under the limit of quantification for IFN-β, IL-1β, and IL-1α, and for IL-6 and TNF-α, there was no significant difference between NTC and *Mda5*-KO macrophages (Supplemental Figure 3).

### Loss of MDA5 restricts the intracellular growth of M. tuberculosis in macrophages with a concomitant reduction in macrophage viability.

To evaluate the role of MDA5 in *M*. *tuberculosis* survival, we evaluated intracellular *M*. *tuberculosis* growth in *Mda5*-KO J774.1 macrophages (Figure 1D). First, we verified a dose-dependent increase in MDA5 protein expression in WT J774.1 macrophages infected with *M*. *tuberculosis* (Figure 1E), similar to previous findings on *Mda5* mRNA expression (27). We found that loss of MDA5 led to a reduction in *M*. *tuberculosis* growth compared with that seen in WT and NTC macrophages (Figure 1F) and that this reduction correlated with a concomitant loss of macrophage viability (Supplemental Figure 4, A and B). This containment and concomitant viability loss were seen only in IFN-γ–primed but not resting macrophages, suggesting that MDA5-mediated host evasion during *M*. *tuberculosis* infection is dependent on the activation status of the macrophage (Figure 1, F and G, and Supplemental Figure 4, A and B). These data show that *M*. *tuberculosis* infection upregulates MDA5 expression and that this MDA5 signaling correlates with permissive *M*. *tuberculosis* proliferation in activated macrophages. In contrast, loss of MDA5 is associated with restriction of *M*. *tuberculosis* proliferation with concomitant macrophage cell death.

### M. tuberculosis infection triggers IL-1β production in an MDA5-dependent manner.

**Figure 2:**
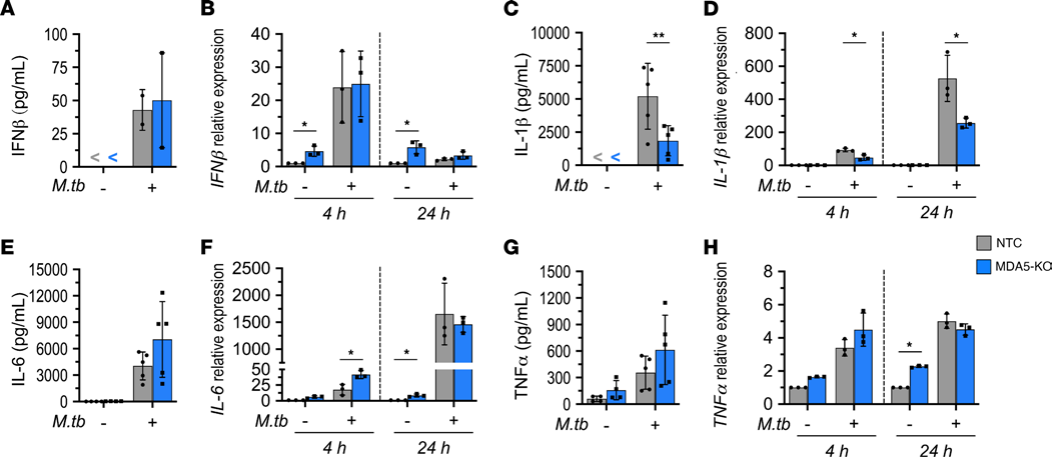
MDA5 is required for IL-1β but not IFN-β production by activated macrophages infected with *M*. *tuberculosis*. *Mda5*-KO and NTC IFN-γ–primed J774.1 cells were infected with *M*. *tuberculosis* (MOI of 1:5). Protein concentration in the culture supernatant and RNA levels of IFN-β (**A** and **B**), IL-1β (**C** and **D**), IL-6 (**E** and **F**), and TNF-α (**G** and **H**) at 4 hours and 24 hours after infection were quantified by Luminex multiplex immunoassay (mean ± SD, *n* = 5 biological replicates from 4 independent experiments; “<” indicates below the limit of quantification) and reverse transcriptase qPCR presented as relative expression to NTC no-infection control (mean ± SD, *n* = 3 biological replicates from 3 independent experiments). *P < 0.05, **P < 0.01 by 2-way ANOVA with Tukey’s posttest.

RLR engagement can lead to induction of type I IFN expression, NF-κB signaling, and inflammasome activation, each of which has been shown to influence the balance between *M*. *tuberculosis* survival and host cells (28–30). To evaluate whether these mechanisms contribute the MDA5-mediated promotion of *M*. *tuberculosis* intracellular growth, we first evaluated the expression patterns of proinflammatory cytokines in IFN-γ–primed *Mda5*-KO macrophages during *M*. *tuberculosis* infection. While we found increased IFN-β levels in response to *M*. *tuberculosis* infection, we did not observe a significant difference in IFN-β protein release or RNA levels between IFN-γ–primed *Mda5*-KO and NTC J774.1 macrophages infected with *M*. *tuberculosis* (Figure 2, A and B). We verified this finding by monitoring IRF activation in response to *M*. *tuberculosis* infection in RAW-Lucia-ISG (WT, *Mda5*-KO, and *RigI*-KO) macrophages that stably express an IRF-inducible Lucia luciferase reporter. In agreement with earlier studies demonstrating a RIG-I–dependent contribution to the type I IFN response during *M*. *tuberculosis* infection (16–18), the RAW-Lucia-ISG-*RigI*-KO reporter macrophages showed reduced IRF activity compared with WT macrophages infected with *M*. *tuberculosis*. In contrast, RAW-Lucia-ISG-*Mda5*-KO cells exhibited no difference in *M*. *tuberculosis*–mediated IRF activity relative to WT cells (Supplemental Figure 5). Considering the fact that *Mda5*-KO cells show diminished IFN-β production in response to transfected *M*. *tuberculosis* RNA (Figure 1C) but not *M*. *tuberculosis* infection (Figure 2, A and B, and Supplemental Figure 5), MDA5-mediated IFN-β production may serve as a compensatory pathway that is masked by the DNA/STING and RNA/RIG-I type I IFN signaling axes triggered during *M*. *tuberculosis* infection.

In line with our observation that MDA5 signaling promotes IL-1β production in response to *M*. *tuberculosis* RNA transfection (Figure 1C), IFN-γ–primed *Mda5*-KO macrophages infected with *M*. *tuberculosis* exhibited a significant reduction in IL-1β secretion (Figure 2C) and RNA levels at both early (4 hours) and later (24 hours) times after infection (Figure 2D). IL-6 expression was similar between *Mda5*-KO and NTC cells at 24 hours after infection (Figure 2, E and F), although *Mda5* knockdown resulted in slightly elevated IL-6 RNA levels at 4 hours after infection (Figure 2F). Similarly, TNF-α expression was not significantly altered between *Mda5*-KO and NTC macrophages following *M*. *tuberculosis* infection (Figure 2, G and H). These observations suggest that the MDA5 signaling response to *M*. *tuberculosis* infection specifically modulates IL-1β production.

### MDA5 plays a role in inflammasome activation during M. tuberculosis infection.

**Figure 3:**
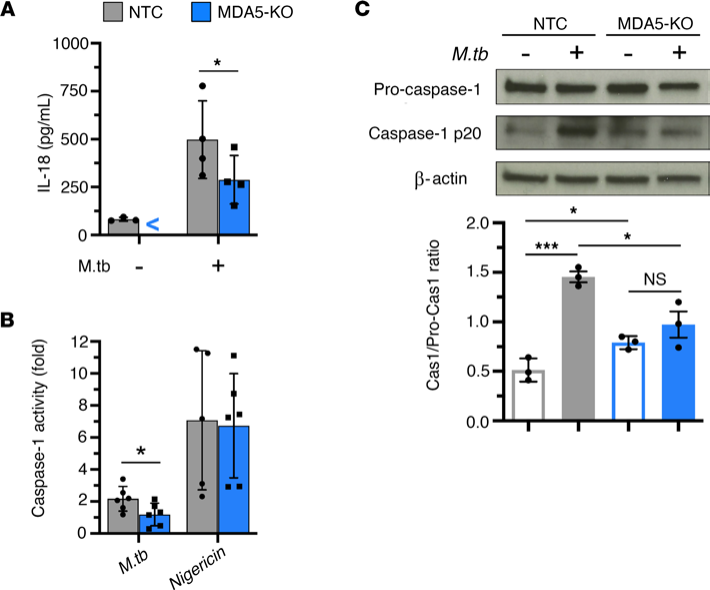
Inflammasome activation in activated macrophages infected with *M*. *tuberculosis* involves MDA5. IFN-γ–primed *Mda5*-KO and NTC J774.1 cells were infected with *M*. *tuberculosis* (MOI of 1:5). (**A**) Level of secreted IL-18 in the cell culture supernatant at 24 hours after infection was quantified by Luminex multiplex immunoassay (mean ± SD, *n* = 4 biological replicates from 3 independent experiments; “<” indicates below the limit of quantification). (**B**) Caspase-1 enzymatic activity at 6 hours after infection measured by cleavage of a luminogenic caspase-1 substrate, Z-WEHD-aminoluciferin (Promega), presented as fold-change relative to no-infection control (mean ± SD, *n* = 6 biological replicates from 4 independent experiments). (**C**) Cleaved caspase-1 and pro–caspase-1 protein levels at 6 hours after infection detected by immunoblot analysis and quantified by densitometry (mean ± SD, *n* = 3 biological replicates from 3 independent experiments). *P < 0.05, ***P < 0.001 by 2-tailed parametric unpaired *t* test.

Considering that RLR sensing of cytosolic *Listeria* *monocytogenes* triggers IL-1β release and inflammasome activation (25), the marked reduction of secreted IL-1β protein in *M*. *tuberculosis*–infected *Mda5*-KO macrophages led us to consider whether MDA5 signaling may promote inflammasome activation during *M*. *tuberculosis* infection. Since inflammasome activation is associated with processing of pro–IL-18 as well as pro–IL-1β, we measured the levels of IL-18 protein secreted by IFN-γ–primed macrophages after 24 hours of *M*. *tuberculosis* infection. As we observed for IL-1β (Figure 2C), induction of mature IL-18 secretion in response to *M*. *tuberculosis* infection was significantly reduced in *Mda5*-KO cells compared with NTC cells (Figure 3A). The immunoassay used for IL-18 quantification (ProcartaPlex Mouse IL-18 Simplex, Thermo Fisher Scientific) is indicated as specific for detecting only the mature form of IL-18, with no cross-reactivity with the pro-form. We then measured caspase-1 enzyme activity as a direct measure of inflammasome activation in macrophages either in response to *M*. *tuberculosis* infection or following treatment with nigericin, a potassium efflux inhibitor known to directly stimulate NLRP3 complex formation to an active inflammasome. We observed that caspase-1 activity following *M*. *tuberculosis* infection was 2-fold lower in activated MDA5-KO macrophages than in NTC control cells (Figure 3B). This decreased inflammasome activity in *M*. *tuberculosis*–infected *Mda5*-KO macrophages was not due to compromise of the inflammasome, since stimulation by nigericin yielded equivalent levels of caspase-1 activity in both *Mda5*-KO and NTC macrophages (Figure 3B). Autoproteolysis of pro–caspase-1 initiated by the inflammasome yields caspase-1 p20 and p10 cleavage fragments to form an active species. Proteolytic processing of pro–caspase-1 was induced by *M*. *tuberculosis* infection in NTC but not *Mda5*-KO activated macrophages after 6 hours, as determined by immunoblot detection of the mature 20 kDa subunit of caspase-1 and densitometric quantification expressed as the ratio of cleaved caspase-1 to pro–caspase-1 (Figure 3C). While *M*. *tuberculosis* infection did not increase caspase-1 p20 in *Mda5*-KO macrophages, basal levels of proteolysis of pro–caspase-1 were significantly higher in activated *Mda5*-KO macrophages than in NTC (Figure 3C). These results reveal that MDA5 plays a role in inflammasome activation during *M*. *tuberculosis* infection. The fact that nigericin stimulation yielded full activity in *Mda5*-KO cells suggests that MDA5-mediated inflammasome activation during *M*. *tuberculosis* infection is via a noncanonical pathway. Importantly, inflammasome signaling has recently been shown to promote *M*. *tuberculosis* survival (31).

### MDA5-deficient macrophages show increased autophagic targeting of intracellular M. tuberculosis.

**Figure 4:**
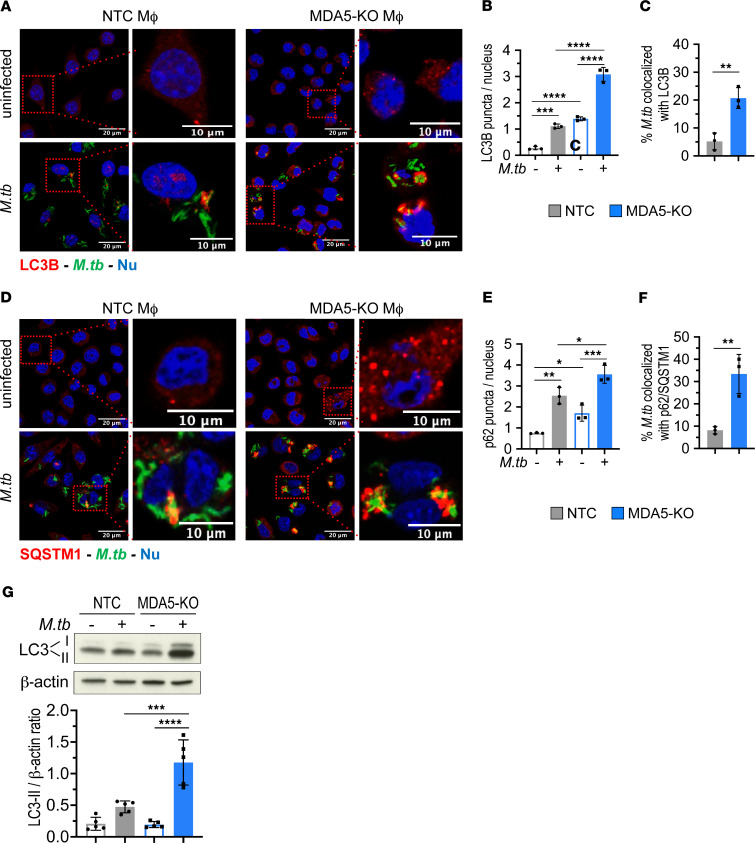
Elevated autophagic flux and targeting of intracellular *M*. *tuberculosis* in *Mda5*-deficient, IFN-γ–primed macrophages. (**A**–**F**) IFN-γ–primed *Mda5*-KO and NTC J774.1 cells were infected with *M*. *tuberculosis* expressing GFP on an episomal plasmid (MOI of 1:10) for 6 hours. (**A**) Fluorescence confocal images of fixed cells immunostained for LC3 (Alexa Fluor 647, far-red) or nuclei (Hoechst 33342, blue). (**B** and **C**) Quantification of LC3 puncta per nucleus (**B**) and *M*. *tuberculosis* colocalization with LC3B (**C**). (**D**) Fluorescence confocal images of fixed cells immunostained for p62 (Alexa Fluor 647, far-red) or nuclei (Hoechst 33342, blue). (**E** and **F**) Quantification of p62 puncta per nucleus (**E**) and *M*. *tuberculosis* colocalization with p62 (**F**). Scale bars: 20 μm for ×63 images. Perinuclear LC3 and p62 puncta were counted in a minimum of 200 cells across different fields. Image processing and analysis were performed using Fiji and Imaris 9.7 software. Data are mean ± SD (*n* = 3 biological replicates from 2 independent experiments). (**G**) LC3-I, LC3-II, and β-actin protein levels in IFN-γ–primed *Mda5*-KO and NTC J774.1 cells infected with *M*. *tuberculosis* (MOI of 1:5) for 24 hours detected by immunoblot analysis. Densitometric quantification of the LC3-II/β-actin ratio presented as the mean ± SD (*n* = 4 biological replicates from 4 independent experiments). *P < 0.05, **P < 0.01, ***P < 0.001, ****P < 0.0001 by 2-way ANOVA with Tukey’s posttest.

Autophagy is a key host defense mechanism for clearance of intracellular pathogens (32), and *M*. *tuberculosis* has developed several strategies to counteract autophagy to ensure its survival (33–36). In light of recent findings linking RLR signaling cascades to autophagy (37–39) and our own data that IFN-γ–primed *Mda5*-KO macrophages restricted *M*. *tuberculosis* proliferation with concomitant loss of cell viability (Figure 1F and Supplemental Figure 4B), we examined autophagic targeting of intracellular *M*. *tuberculosis* bacilli in MDA5-KO and NTC macrophages following *M*. *tuberculosis* infection. Immunofluorescence imaging of the autophagosome membrane-specific marker microtubule-associated light chain 3 (LC3) revealed significantly higher LC3 puncta formation in IFN-γ–primed *Mda5*-KO macrophages as early as 6 hours after *M*. *tuberculosis* infection compared with NTC cells (Figure 4, A and B). In addition, we found a robust increase in *M*. *tuberculosis* and LC3 colocalization in IFN-γ–primed *Mda5*-KO macrophages compared with NTC controls (Figure 4C), suggesting efficient autophagic targeting of intracellular bacilli. In light of a previous report indicating that the delivery of *M*. *tuberculosis* bacilli to autophagosomes requires the autophagy receptor p62 (7), we examined whether the enhanced autophagic targeting of intracellular *M*. *tuberculosis* in *Mda5*-KO cells involves p62. IFN-γ–primed *Mda5*-KO macrophages showed increased levels of p62 puncta per cell in both uninfected and *M*. *tuberculosis*–infected macrophages compared with NTC cells (Figure 4, D and E). *Mda5* KO also resulted in a significant increase in *M*. *tuberculosis* bacilli colocalization with p62 puncta (Figure 4F). LC3 and p62 puncta in uninfected MDA5-KO cells were also slightly elevated relative to NTC cells, indicating that the loss of MDA5 increases basal autophagy levels in IFN-γ–primed uninfected macrophages (Figure 4, B and E). Immunoblot analysis of LC3-II protein levels also showed significantly increased accumulation of LC3-II in *M*. *tuberculosis*–infected MDA5-KO cells compared with NTC controls (Figure 4G). Moreover, the inhibition of autophagic flux by treatment with bafilomycin A1, a potent inhibitor of the vacuolar H^+^ATPase, significantly increased the LC3-II lipidation in MDA5-KO cells, verifying that the accumulation of LC3-II in the absence of MDA5 was not due to the suppression of autophagic flux (Supplemental Figure 6). These data demonstrate that during *M*. *tuberculosis* infection in macrophages, MDA5 signaling leads to reduced autophagic targeting of the pathogen, supporting an MDA5-mediated subversion mechanism by which *M*. *tuberculosis* inhibits host immune destruction.

### MDA5 facilitates M. tuberculosis survival in mice.

**Figure 5:**
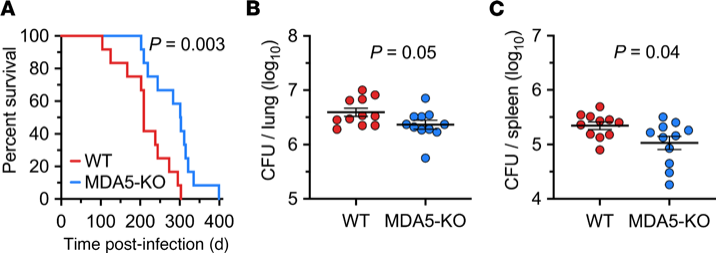
*Mda5*-deficient mice show increased resistance to infection with *M*. *tuberculosis*. (**A**) Survival of *Mda5^−/−^* and WT C57BL/6 mice after low-dose aerosol infection with the *M*. *tuberculosis* H37Rv strain (*n* = 12 per group). Both mouse strains were infected in the same chamber simultaneously, and day 1 lung CFU counts revealed an implantation of 2.1 log_10_ CFU. *P* < 0.01 by log-rank (Mantel-Cox) test. (**B** and **C**) Bacillary burdens shown as log_10_ CFU per organ in lung (**B**) and spleen (**C**) of *Mda5^−/−^* and WT C57BL/6 mice at 4 weeks after aerosol infection. Data are mean ± SEM (*n* = 11 per group). *P < 0.05 by 2-tailed parametric unpaired *t* test.

To extend our in vitro findings, we evaluated the role of MDA5 in *M*. *tuberculosis* pathogenesis using a mouse model of *M*. *tuberculosis* infection. We infected WT and congenic MDA5^–/–^ C57BL/6 mice by low-dose aerosol infection. Day 1 bacillary burden in infected lungs revealed equal implantation (~2.1 log_10_ CFU) of *M*. *tuberculosis* in both mouse strains (Supplemental Table 1). Loss of MDA5 conferred a significant increase in the survival of *M*. *tuberculosis*–infected animals compared with WT mice (median time to death of 302 days and 209 days, respectively) (Figure 5A). *Mda5^–/–^* mice had reduced lung and spleen bacillary loads compared with WT control mice at 4 weeks after infection (Figure 5, B and C). We also evaluated protein levels of IL-18, IL-1β, IL-1α, IL-6, and TNF-α in whole lung tissue from *Mda5^−/−^* and WT C57BL/6 mice 12 weeks after *M*. *tuberculosis* infection, and RNA levels of IFN-β, IL-1β, and IL-6 at 4 weeks after infection (Supplemental Figure 7, A–I), but did not find a statistically significant difference in the expression of these cytokines, except for IL-18, which was undetectable in 3 of 6 *Mda5^−/−^* mice compared with WT mice, and IL-1β, which was elevated in 3 of 6 *Mda5^−/−^* mice. This is in keeping with the study by Manzanillo et al., who reported an increase in IL-1β 3 weeks after infection in the serum of mice that lacked IRF3 (a transcription factor activated downstream following pathogen recognition by RNA sensors), but in contrast with the study by Cheng and Schorey, who found a RIG-I–dependent decrease in IFN-β in alveolar macrophages from mice that lacked the downstream signaling adaptor MAVS 2 weeks after *M*. *tuberculosis* infection (16, 20). The differences could be related to examining whole lung versus alveolar macrophages, to early and late time points after infection, or to specific RNA sensors involved. Nevertheless, our studies show that loss of MDA5 is protective for mice during *M*. *tuberculosis* infection similar to that observed in MAVS^–/–^ and IRF3^–/–^ mice (16, 20), although MAVS^–/–^ and IRF3^–/–^ mice show more significant increase in survival following *M*. *tuberculosis* infection compared with MDA5^–/–^ mice. A potential explanation for this observation is that MAVS is a common signaling adaptor molecule for both RIG-I and MDA5, and their interaction further activates IRF3 among other transcription factors. Therefore, the enhanced survival in MAVS^–/–^ and IRF3^–/–^ mice could be the consequence of combined loss of RIG-I and MDA5 signaling.

### M. tuberculosis sensing in human macrophages modulates MDA5 activation.

**Figure 6:**
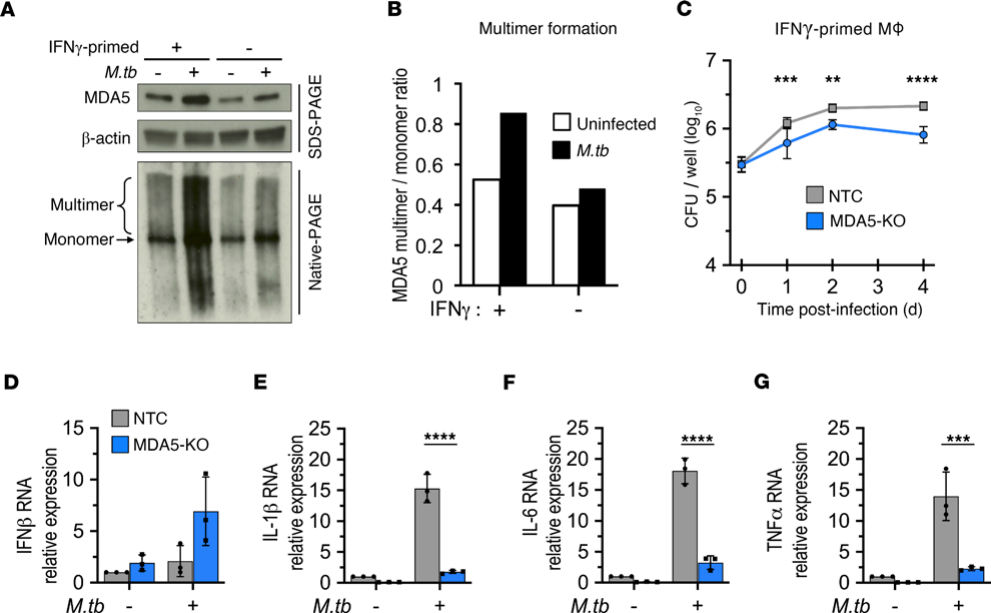
MDA5 is activated during *M*. *tuberculosis* infection and promotes intracellular bacillary growth in primed human macrophages. (**A**) IFN-γ–primed or resting human monocyte-derived macrophages were infected with *M*. *tuberculosis* (MOI of 1:10) for 24 hours. Whole-cell lysates were subjected to native or SDS-PAGE followed by immunoblot analysis. Data are representative of 1 independent experiment. (**B**) Densitometric analysis was used to quantify band intensities in **A** presented as a ratio of MDA5 multimer to monomer. (**C**) Growth kinetics of *M*. *tuberculosis* (MOI of 1:5) in PMA-differentiated and IFN-γ–primed THP-1 cells (NTC and *MDA5*-KO). Data are the mean CFU ± SD for each time point (*n* = 6 biological replicates from 2 independent experiments). (**D**–**G**) PMA-differentiated and IFN-γ–primed THP-1 cells (NTC and *MDA5*-KO) were infected with *M*. *tuberculosis* (MOI of 1:5) for 24 hours. RNA levels of IFN-β (**D**), IL-1β (**E**), IL-6 (**F**), and TNF-α (**G**) were measured by reverse transcriptase qPCR and presented as fold-change relative to NTC no-infection control. RNA levels were normalized to PolR2A expression (mean ± SD, *n* = 3 biological replicates from 3 independent experiments). **P < 0.01, ***P < 0.001, ****P < 0.0001 by 2-way ANOVA with Tukey’s posttest.

We next evaluated whether *M*. *tuberculosis* also triggers MDA5 signaling in human macrophages. We found that MDA5 levels were elevated during *M*. *tuberculosis* infection of hMDMs, similar to increases seen in mouse macrophages (Figure 1E), and this effect was more pronounced in IFN-γ–primed macrophages (Figure 6A, top). RLRs are known to undergo ligand-triggered oligomerization leading to active signaling assemblies. Native PAGE revealed that *M*. *tuberculosis* infection substantially promoted MDA5 oligomerization in hMDMs compared with uninfected controls (Figure 6A). The ratio of MDA5 multimers to monomer was greater in IFN-γ–stimulated hMDMs than in resting cells upon *M*. *tuberculosis* infection (Figure 6B), verifying that MDA5 activation was amplified by IFN-γ macrophage activation. We had observed oligomerization of RIG-I in murine J774 cells but due to technical difficulties could not establish oligomerization of MDA5 in either J774 cells or murine bone marrow–derived macrophages. To verify that our murine macrophage experiments accurately reflect the human phenotype, we infected *MDA5*-KO and WT THP-1 macrophages (KO validation shown in Supplemental Figure 2C) with *M*. *tuberculosis* and monitored bacterial proliferation. Consistent with our findings in J774 macrophages (Figure 1G), IFN-γ–primed THP-1 *MDA5*-KO macrophages displayed a significantly greater ability to restrict *M*. *tuberculosis* intracellular proliferation than NTC cells (Figure 6C), and this was accompanied by concomitant increased cell death in the *MDA5*-KO macrophages compared with the NTC controls (Supplemental Figure 8). We again found that MDA5 was required for IL-1β production (Figure 6E) but not IFN-β (Figure 6D) in human macrophages, similar to our observations in mouse macrophages (Figure 2), a notable difference being that unlike the J774 mouse cells (Figure 2, E–H), the expression of IL-6 and TNF-α in human THP-1 macrophages was significantly reduced in MDA5-KO cells (Figure 6, F and G). This could simply be a species difference or an effect of phorbol 12-myristate 13-acetate (PMA) used for THP-1 monocyte differentiation. More importantly, the strengths of the responses of primary mouse macrophages and mouse cell lines such as J774 to *M*. *tuberculosis* infection have been reported to be considerably different from each other, while THP-1 cells show similar responses to *M*. *tuberculosis* infection compared to primary human macrophages (27, 40).

## Discussion

**Figure 7:**
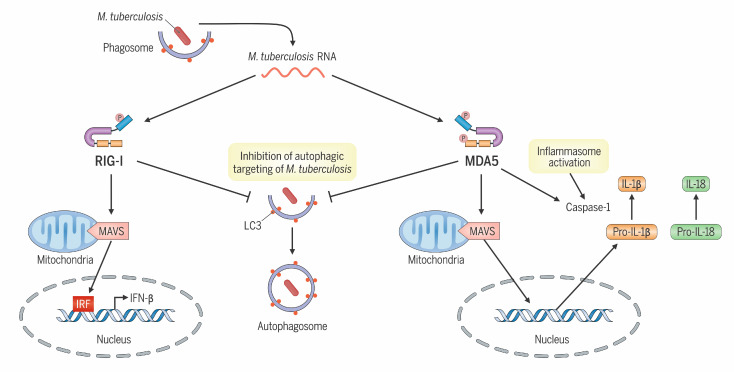
RIG-I–like receptor sensing of *M*. *tuberculosis* RNA promotes pathogen survival. MDA5 activation facilitates *M*. *tuberculosis* survival through a culmination of skewed proinflammatory cytokine production and inflammasome activation and attenuation of autophagic pathways. RIG-I sensing of *M*. *tuberculosis* RNA contributes to *M*. *tuberculosis*–mediated type I IFN responses and innate immune subversion though decreased autophagic responses.

The interplay between *M*. *tuberculosis* and cytosolic nucleic acid–sensing pathways has emerged as a key battleground in TB pathogenesis, revealing vital immune subversion strategies employed by *M*. *tuberculosis* and host cell countermeasures (5–8, 16–18, 20). Cyclic dinucleotides and dsDNA released by *M*. *tuberculosis* have been well established as activators of the STING pathway that simultaneously lead to both induction of type I IFNs and autophagy, which, in sum, elicit a net positive host benefit. However, the influence of cytosolic *M*. *tuberculosis* RNA on bacterial pathogenesis has just begun to be explored. Herein, our data reveal a distinct role for the RNA sensor MDA5 within a complex network of nucleic acid–sensing pathways, promoting *M*. *tuberculosis* survival against the suppressive antimicrobial forces activated in IFN-γ–primed macrophages through a mechanism involving skewed IL-1β production, inflammasome activation, and attenuation of selective autophagic targeting of *M*. *tuberculosis* (Figure 7). The supportive role of MDA5 in primed macrophages for *M*. *tuberculosis* survival could be especially relevant during early infection, when IFN-γ levels are known to increase in bronchoalveolar lavage fluid, and *M*. *tuberculosis*–infected IFN-γ–primed alveolar macrophages (AMs) in the lung skew toward M1 polarization, which is less permissive for the growth of the pathogen (41).

Considering that a variety of molecularly diverse mycobacterial pathogen-associated molecular patterns (PAMPs) are known to trigger host response signaling cascades in infected macrophages, we used a macrophage *M*. *tuberculosis* RNA transfection assay to characterize *M*. *tuberculosis* RNA activation of the cytosolic innate immune sensors and uncover host factors driving an innate response to this microbial PAMP. Similar to the previously demonstrated findings with RNA derived from Gram-positive and Gram-negative intracellular bacteria (26, 42–45), we observed that cytosolic delivery of *M*. *tuberculosis* RNA not only induces type I IFN production but also triggers a proinflammatory cytokine signature in macrophages, suggesting that *M*. *tuberculosis* RNA has intrinsic immunogenic properties and is capable of stimulating cytosolic sensors. However, our data do not prove that specific *M*. *tuberculosis* RNA species are detected by MDA5 during infection. Cheng and Schorey identified specific *M*. *tuberculosis* RNA transcripts, *M*. *tuberculosis* mRNAs, *mce1B*, *rpoC*, and *ppe11*, in the cytosol of cells infected with *M*. *tuberculosis* that amplify IFN-β production through the RIG-I RNA-sensing pathway (16). However, the full scope of *M*. *tuberculosis* RNA species capable of triggering the cytosolic innate immune sensors remains undefined, and the question of whether the same or different *M*. *tuberculosis* RNA species are recognized by RIG-I versus MDA5 must still be addressed. Considering that MDA5 has been shown to interact with host mitochondrial dsRNA (46), and that *M*. *tuberculosis* infection causes mitochondrial membrane disruption (47), it remains plausible that host RNAs may also engage MDA5 during *M*. *tuberculosis* infection.

Increased MDA5 gene expression is often used as a proxy indicator of MDA5 activation, but specific activation occurs through protein oligomerization and filament formation around long dsRNA. Here we show that *M*. *tuberculosis* infection not only induced MDA5 protein expression (Figure 1E) but also triggered the formation of active multimeric species in human macrophages, which was further enhanced in IFN-γ–primed macrophages (Figure 6, A and B). In addition, we observed that loss of MDA5 restricted intracellular *M*. *tuberculosis* proliferation in both human and mouse IFN-γ–primed macrophages to a greater degree than seen in control macrophages. Interestingly, an earlier study by Ranjbar et al. reported that MDA5 proficiency restricted intracellular *M*. *tuberculosis* growth in human THP-1 monocyte cells (17). This discrepancy between our observations and those by Ranjbar et al. may be related to differences in the activation state of the infected macrophages or to different THP-1 differentiation methods used. We used differentiated, IFN-γ–primed THP-1 cells that more closely resemble AMs obtained by differentiation of bone marrow–derived monocytes, priming of which in vivo requires T cell–derived IFN-γ (48), while Ranjbar et al. used undifferentiated unprimed THP-1 cells. Indeed, our study reveals that differences between NTC and MDA5 macrophages in *M*. *tuberculosis* containment were only observed in cells that have been IFN-γ–primed (Figure 1, F and G). It is hypothesized that most of the lung innate immune responses derive from recruitment or changes in monocyte-derived AMs, unlike resident AMs, which are terminally differentiated and are less immunoreactive (49). In addition, our data, showing that loss of MDA5 enhanced host *M*. *tuberculosis* containment both in vitro and in vivo, are consistent with the work of Cheng and Schorey, who found that *M*. *tuberculosis*–infected mice lacking the MDA5 downstream signaling adaptor MAVS also demonstrate greater *M*. *tuberculosis* containment (16). A limitation of our study is that while we showed improved containment of *M*. *tuberculosis* growth in MDA5-KO human THP-1 cells, we were unable to confirm this same phenotype in primary murine bone marrow–derived macrophages (BMDMs) from MDA5^–/–^ mice due to technical issues with the viability of these BMDMs following IFN-γ priming and *M*. *tuberculosis* infection.

A key finding in our study is that 2 important innate immune responses during early-stage *M*. *tuberculosis* infection — IL-1β production and inflammasome activation — are MDA5 dependent. Interestingly, we observed that MDA5 was not essential for the induction of IFN-β production or IRF activation by macrophages during *M*. *tuberculosis* infection, as shown in 3 MDA5-KO macrophage cell lines that were independently engineered: mouse J774.1 and RAW-Lucia-ISG cells (Figure 2, A and B, and Supplemental Figure 3) and PMA-differentiated human THP-1 cells (Figure 6D). Hence, in WT macrophages infected with *M*. *tuberculosis*, the robust type I IFN responses observed appear to be driven primarily by cGAS, STING, and RIG-I signaling, while PAMPs recognized by MDA5 appear to trigger IL-1β production and inflammasome activation. Our data indicating a type I IFN–independent role for MDA5 in the innate immune response to *M*. *tuberculosis* are consistent with previous reports demonstrating a type I IFN–independent function for MDA5 in other disease states, namely the antiviral response to Sendai virus infection (50) and the mitochondrial apoptosis pathway in melanoma cells (51).

While our data suggest that MDA5 signaling modulates a network of innate immune mechanisms in macrophages infected with *M*. *tuberculosis*, our findings do not decipher the relative contribution of MDA5-mediated inhibition of autophagy, in comparison with its impact on the dysregulation of IL-1β production and inflammasome activation, in promoting *M*. *tuberculosis* survival. Activation of MDA5 during *M*. *tuberculosis* infection may amplify crosstalk between inflammasome signaling and autophagy (52), which has recently come to light as necessary balance for immune homeostasis (53). The NLRP3–caspase-1 inflammasome has previously been shown to negatively regulate autophagy in a stimulus-dependent manner (54–57), in line with our data demonstrating that MDA5 signaling during *M*. *tuberculosis* infection both elevates inflammasome activation (Figure 3) and dampens autophagic flux (Figure 4) in response to the infection. Additionally, the interplay between RLRs and autophagic processes remains unclear, and recent studies have shown both elevation and attenuation of autophagy by RIG-I or MDA5 antiviral signaling, respectively (38, 39, 53). Further, a critical role for canonical autophagy in containing *M*. *tuberculosis* infection is still not fully defined. *M*. *tuberculosis* impairment of cell autophagic flux in macrophages has been demonstrated in vitro (58), yet other studies have shown that loss of genes that promote autophagy does not always lead to susceptibility to *M*. *tuberculosis* infection (59, 60). This could either be reflective of the highly effective nature of inhibitors of autophagy produced by *M*. *tuberculosis* or of a noncanonical, autophagy-independent role played by key autophagy factors such as autophagy related 5 protein in containing *M*. *tuberculosis*–mediated pathology (60). In addition, as part of its host immune defense role, IFN-γ is known to modulate autophagy and expression of proinflammatory cytokines in macrophages (61, 62). Our findings of increased LC3-II and p62 puncta in uninfected MDA5-KO cells over NTC (Figure 4B), and a decrease in IL-1β, IL-6, and TNF-α in uninfected *MDA5*-KO cells over NTC (Figure 6, E–G), suggest that MDA5 might have a role in modulating IFN-γ signaling not specific to *M*. *tuberculosis* infection, and this could have broader implications for other bacterial and viral infections.

In summary, our study reveals that MDA5 is activated during *M*. *tuberculosis* infection and that in MDA5-KO cells and animals there are reduced IL-1β expression, reduced inflammasome activation, and increased autophagy. Thus, in the WT, *M*. *tuberculosis* appears to subvert MDA5 signaling in a manner that enhances its ability to proliferate both in vitro *and* in vivo. Further elucidation of the mechanisms by which the RLR family of RNA sensors are exploited by *M*. *tuberculosis* may reveal molecular targets for novel TB host-directed therapeutic strategies.

## Methods

### Bacterial strains.

*M*. *tuberculosis* strains (H37Rv, CDC1551, and CDC1551 expressing a P_hsp60_:gfp episomal plasmid) (63) were obtained from the Johns Hopkins Center for Tuberculosis Research and previously authenticated by deep sequencing, confirming intact PDIM biosynthetic genes (64). Stocks were grown to an OD_600_ value of 1 in Middlebrook 7H9 broth supplemented with 10% (vol/vol) oleic acid–albumin–dextrose–catalase (OADC; Difco), 0.5% (vol/vol) glycerol, and 0.05% (vol/vol) Tween 80 and stored in 20% (vol/vol) glycerol at –80°C.

### Cell culture.

Murine J774.1 macrophage cells and human THP-1 monocytes (ATCC) were cultured in RPMI-GlutaMAX Medium (Gibco) supplemented with 10% (vol/vol) heat-inactivated FBS (Gibco). THP-1 cells were differentiated into macrophage-like cells by incubation with PMA at a concentration of 20 μM for 24 hours, after which the medium was removed and replaced with fresh complete culture medium for another 24 hours before use for in vitro experiments. The RAW 264.7–derived macrophages RAW-Lucia-ISG, RAW-Lucia-ISG-MDA5-KO, and RAW-Lucia-ISG-RIGI-KO (InvivoGen) were cultured according to the manufacturer’s protocol in DMEM, high glucose, and GlutaMAX (Gibco) supplemented with 10% (vol/vol) heat-inactivated FBS (Gibco), 100 μg/mL Normocin (InvivoGen), and 200 μg/mL Zeocin (InvivoGen). All cell lines are free of mycoplasma contamination. Macrophage IFN-γ priming was performed with 100 ng/mL IFN-γ for 16–20 hours prior to infection.

For hMDM isolation and generation, human PBMCs were purified from deidentified whole blood obtained from Anne Arundel Medical Blood Donor Center (Anne Arundel, Maryland, USA) using Ficoll-Paque PLUS (GE Healthcare) density centrifugation. Total PBMCs were then seeded in non–tissue culture–treated flasks and incubated in RPMI-GlutaMAX supplemented with 10% (vol/vol) FBS, 1% (vol/vol) penicillin/streptomycin (Gibco), and 25 ng/mL macrophage colony-stimulating factor (M-CSF) (PeproTech) for a total of 6 days. After the first 24 hours, nonadherent cells were removed by gentle washes with PBS, and the adherent monocyte cells were differentiated by further culture in complete medium plus M-CSF. Two days before infection experiments, the hMDMs were trypsinized, counted, and reseeded in cell culture plates dictated by the specific in vitro experiment.

### Mice.

Six-week-old female WT C57BL/6 mice (strain 000664) and MDA5^–/–^ mice on a C57BL/6 background (strain 015812) were purchased from The Jackson Laboratory and housed within the institutional animal facility. Mice were maintained under animal biosafety level 3 conditions on a 12-hour light/12-hour dark cycle, with rodent chow and water available ad libitum. All animal procedures were approved by the Institutional Animal Care and Use Committee of the Johns Hopkins University School of Medicine.

### MDA5-KO cells.

The J774.1 macrophage MDA5-KO clonal cell line was engineered by CRISPR/Cas9 nuclease RNA-guided genome editing using the pLV-U6g-EPCG lentiviral system (Sigma-Aldrich, Target ID MM0000161076) as previously described (18). Lentiviral transduction was performed as previously described (65). Briefly, HEK293T cells were transfected with the pLV-U6g-EPCG vector (guide RNA [gRNA], and Cas9 elements flanked by puromycin and GFP) and the packaging vectors pCMVR8.91 and pVSVg to produce lentivirus particles. After 72 hours, culture supernatant was centrifuged for 200*g* for 10 minutes at room temperature and passed through a 0.45 μm filter. J774.1 macrophages were inoculated with the lentivirus-containing supernatant along with Polybrene (Sigma-Aldrich) at a concentration of 8 mg/mL for 24 hours and then washed twice with PBS and cultured for an additional 48 hours in complete RPMI medium. The cells were then treated with puromycin (Sigma-Aldrich) at a concentration of 2 mg/mL for 4 days, followed by selection of single-cell clones expressing gRNA/Cas9 through FACS for GFP-positive cells. A CRISPR/Cas9 nontarget cell line was generated in parallel as a control for off-target DNA mutagenesis and cellular passage number (Sigma-Aldrich). KO was verified by immunoblot analysis of MDA5 protein expression, and gene mutagenesis was verified by next-generation sequencing (GENEWIZ).

A THP-1 MDA5-KO clonal cell line was purchased from Synthego and, in brief, was engineered by CRISPR/Cas9 nuclease RNA-guided genome editing using transfection of a validated sgRNA and single-cell selection. Synthego confirmed KO by sequence verification of alleles. MDA5 KO was further verified by immunoblot analysis.

### RNA isolation.

For isolation of total *M*. *tuberculosis* RNA, the *M*. *tuberculosis* H37Rv strain was grown to mid-log phase in 50 mL of culture medium, described above, centrifuged at 5,000*g* for 5 minutes at room temperature, and washed with PBS twice. The *M*. *tuberculosis* pellet was then lysed with 1 mL TRIzol (Invitrogen). Cells were disrupted using the Precellys Evolution homogenizer at frequency 7,400 beats per minute for 30 seconds for 3 cycles, with a 1-minute incubation on ice between each cycle. For isolation of total human RNA, THP-1 cells were grown to 1 × 10^6^ cells per mL, centrifuged at 200*g* for 5 minutes, and washed once with PBS. The THP-1 cell pellet was then lysed with 1 mL TRIzol. Total RNA from both *M*. *tuberculosis* and THP-1 cells lysed in TRIzol was then isolated using phenol-chloroform extraction and isopropanol precipitation as described in the TRIzol reagent manufacturer’s protocol. DNase digest was performed using TURBO DNase (Ambion), and RNA was subsequently re-extracted using acid-phenol:chloroform (pH 4.5; Ambion), per the manufacturer’s protocol. The ratio of UV absorbance at 260 and 230 nm was used a secondary measure of nucleic acid purity. The abundance of RNA relative to any trace genomic DNA contaminants in the RNA extracts was confirmed by qPCR for 5S and 16S rRNA transcripts using a no–reverse transcriptase (NRT) control. 16S rRNA transcripts were detected using the following primers: forward (5′→3′) CCTGGGAAACTGGGTCTAATAC, reverse (5′→3′) CTCATCCCACACCGCTAAA, and probe (5′→3′) FAM-ATGCATGTCTTGTGGTGGAAAGCG-MGB. 5S rRNA transcripts were detected using the following primers: forward (5′→3′) CACAGCGGCAGGGAAAC, reverse (5′→3′) TTCGGCGGTGTCCTACTT, and probe (5′→3′) FAM-CCCATTCCGAACCCGGAAGCTAAG-MGB.

### RNA transfection.

J774.1 macrophages (resting or prestimulated with IFN-γ 1 day before infection) or hMDMs were seeded 1 day before transfection. The cells were then transfected with RNA (total *M*. *tuberculosis* RNA, total *M*. *tuberculosis* RNA pretreated with RNaseV, total human RNA, or 5′-ppp-dsRNA) at a final concentration of 1 mg/mL for 1 mg total RNA per well in a 12-well plate using LyoVec (InvivoGen) following the manufacturer’s protocol. For the RNaseV treatment control samples, total *M*. *tuberculosis* RNA was incubated at 30°C with RNaseV (Thermo Fisher Scientific EN0531) at a final concentration of 10 mg/mL for 30 minutes according to the manufacturer’s protocol. The absence of RNA in the RNaseV-treated samples was confirmed by qPCR for 5S and 16S rRNA transcripts using a no–reverse transcriptase (NRT) control. The 5′-ppp-dsRNA was purchased from InvivoGen and prepared per the manufacturer’s protocol. Culture supernatants were collected, and macrophage cellular total RNA was extracted 24 hours after transfection for downstream cytokine protein and RNA transcript analysis as described below.

### M. tuberculosis macrophage infection.

Macrophage cells (J774.1, RAW-Lucia-ISG, hMDM, or PMA-differentiated THP-1) were seeded on cell culture plates (size and number of cells dictated by the specific in vitro assay) in complete macrophage culture medium, and infections were carried out in either resting or IFN-γ–primed cells as previously described (18). For bacterial inoculation, early log-phase cultures of CDC1551 or H37Rv *M*. *tuberculosis* strains were washed twice in PBS, suspended in complete macrophage culture medium (specific for each cell type described above), passed through a 23-gauge needle to break bacterial clumps, and added to the macrophages at the appropriate multiplicity of infection (MOI) for 2 hours. The macrophages were then washed 3 times with RPMI or DMEM to remove extracellular bacteria and further cultured in complete medium as described above for the indicated time.

### Intracellular M. tuberculosis growth assay.

J771.1 or PMA-differentiated THP-1 macrophages, prestimulated with IFN-γ 1 day before infection, were inoculated with the *M*. *tuberculosis* CDC1551 strain at an MOI of 5 for 2 hours and washed with RPMI as described above. Cells were then incubated in complete medium for another 2 hours (day 0) to determine the number of phagocytized bacteria or for another 1, 2, 3, and 4 days to enumerate bacterial growth. At each time point, cells were washed with PBS once and lysed using 0.025% SDS. A series of 10-fold dilutions of the cell lysates were made in PBS and then inoculated on MB7H11 agar plates supplemented with 10% (vol/vol) OADC and 0.2% (vol/vol) glycerol. The plates were incubated at 37°C for 3–4 weeks, after which the number of colonies was counted and calculated to determine log_10_ CFU per cell culture well.

### Cell viability assay.

J771.1 or PMA-differentiated THP-1 macrophages, prestimulated with IFN-γ 1 day before infection, were inoculated with the *M*. *tuberculosis* CDC1551 strain at an MOI of 5 for 2 hours and washed with RPMI as described above. Cells were then incubated in complete medium for 1, 2, 3, and 4 days. At each time point, the medium was replaced with fresh prewarmed complete culture medium to remove *M*. *tuberculosis* present in the cell culture supernatant, and cell viability was evaluated using the CellTiter 96 AQueous One Solution Cell Proliferation Assay (Promega) according to the manufacturer’s instructions.

### Real-time qPCR.

Total RNA from macrophages was extracted at the indicated time points using the RNeasy Mini Kit, including an on-column DNase digest, according to the manufacturer’s protocol (QIAGEN). Complementary DNA (cDNA) synthesis was performed using qScript cDNA SuperMix following the manufacturer’s protocol (Quanta Biosciences). A fraction of the RNA was retained for reverse transcriptase–negative control reactions. Quantitative reverse transcription PCR was performed using TaqMan gene expression assays (IFN-β1: mouse Mm00439552_s1, human Hs01077958_s1; IL-1β: mouse Mm00434228_m1, human Hs01555410_m1; IL-6: mouse Mm00446190_m1, human Hs00174131_m1; TNF-α: mouse Mm00443258_m1, human Hs00174128_m1; PolR2A: mouse Mm00839502_m1, human Hs00172187_m1) with TaqMan Universal PCR Master Mix (Applied Biosystems) according to the manufacturer’s instructions on a StepOnePlus Real-Time PCR system (Applied Biosystems). RNA transcript levels were normalized to PolR2A expression and presented as fold-change relative to uninfected cells as determined by the ΔΔCt method.

### Multiplex cytokine analysis.

Macrophage cell culture supernatants were collected 24 hours after *M*. *tuberculosis* infection and passed through a 0.2 μM filter (Corning Costar Spin-X) to remove cellular debris and bacteria. Milliplex multiplex assays coupled with the Luminex platform (Millipore) were used to measure indicated cytokine analytes.

### IRF activation luciferase reporter assay.

RAW-Lucia-ISG, RAW-Lucia-ISG-MDA5-KO, and RAW-Lucia-ISG-RIGI-KO (InvivoGen) cells were generated from the murine RAW 264.7 macrophage cell line by stable integration of a secreted Lucia luciferase reporter under the control of an ISG54 minimal promoter and 5 IFN-stimulated response elements, thus allowing IRF activation to be monitored by levels of Lucia luciferase in the cell culture supernatant. In brief, the cells were inoculated with the *M*. *tuberculosis* H37Rv strain at an MOI of 5 or 20 for 2 hours and then washed with DMEM and further cultured in complete DMEM. After 24 hours of incubation, supernatants were collected and assayed for the level of IRF induction by determination of the activity of Lucia luciferase using QUANTI-Luc (InvivoGen) according to the manufacturer’s protocol.

### Immunoblot analysis.

Whole-cell lysates were prepared by addition of lysis buffer (20 mM Tris-HCl [pH 7.5], 150 mM NaCl, 1 mM Na_2_EDTA, 1 mM EGTA, 1% Triton, 2.5 mM sodium pyrophosphate, 1 mM β-glycerophosphate, 1 mM Na_3_VO_4_, 1 μg/mL leupeptin; Cell Signaling Technology) to the macrophage cells at predefined time points after infection for 15 minutes on ice. After centrifugation for 10 minutes at 10,000*g*, lysate samples were resolved by SDS-PAGE on a 4%–15% Mini-PROTEAN TGX gel (Bio-Rad), followed by transfer onto a PVDF membrane. The membrane was blocked with TBS containing 0.1% Tween-20 and 5% nonfat dry milk and incubated with the following antibodies: rabbit anti-MDA5 (Cell Signaling Technology 5321; 1:1,000), rabbit anti-LC3 (Cell Signaling Technology 12741; 1:2,000), rat anti–caspase-1 (eBioscience 5B10; 1:1,000), followed by species-matched anti-rabbit or anti-rat IgG-HRP (Cell Signaling Technology 7074 or 7077, respectively; 1:5,000), or mouse anti–β-actin–HRP (Abcam 49900; 1:100,000), followed by species-matched anti-rabbit or anti-rat IgG-HRP (Cell Signaling Technology 7074 or 7077, respectively; 1:5,000). Membranes were washed 3 times with TBS plus 0.1% Tween 20 between each antibody incubation step. ECL Western Blotting Substrate (Pierce) was used to detect labeled proteins, and blots were developed using Amersham Hyperfilm ECL (Cytiva). Densitometry analyses of the immunoblots were carried out with ImageJ software (NIH), ensuring local background subtraction and band intensity of proteins of interest in a linear range on the selected film exposure.

### Native PAGE.

Native PAGE was used as described previously for the analysis of the oligomeric state of MDA5 (66). In brief, resting or IFN-γ primary hMDMs were infected with the *M*. *tuberculosis* CDC1551 strain at an MOI of 10 for 24 hours. The cells were washed once in ice-cold PBS, and whole-cell lysates were prepared by addition of lysis buffer (25 mM Tris-HCl [pH 7.4], 150 mM NaCl, 0.5% Triton X-100, 1 mM PMSF, 1× protease inhibitor cocktail) to the macrophage cells for 30 minutes on ice. After centrifugation for 10 minutes at 10,000*g*, protein concentration for each lysate sample was determined using the Bradford protein assay (Bio-Rad) according to the manufacturer’s protocol and diluted to 1 μg/uL in 1× native sample buffer (Bio-Rad). Lysate samples (input 25 mg protein) were resolved on a 7% Mini-PROTEAN TGX Precast Protein Gel (Bio-Rad) run in Tris/glycine buffer without SDS. The gel was incubated in transfer buffer for 1 minute, and the PVDF membrane was pre-equilibrated in methanol for 1 minute and rinsed in transfer buffer before transfer sandwich assembly, followed by transfer onto a PVDF membrane using Tris/glycine plus 0.04% SDS transfer buffer. The membrane was then probed for MDA5 as described above. Each lysate sample was also resolved using SDS-PAGE, transferred to a PVDF membrane, and probed for MDA5 and β-actin as described above to determine the level of total MDA5 protein expression in each sample.

### Caspase-1 activity assay.

The Caspase-Glo 1 assay (Promega) was used to measure the activity of caspase-1 in J774.1 macrophages either infected with *M*. *tuberculosis* at an MOI of 5 or treated with nigericin, an NLRP3 inflammasome inducer, at a final concentration of 20 μM. After 4–6 hours of infection or nigericin treatment, unprocessed cell samples were mixed at a 1:1 volume ratio with assay reagent in 96-well opaque plates, and the activity of caspase-1 in each sample was measured according to the manufacturer’s protocol. In brief, after 90 minutes of incubation at room temperature, luminescence produced by caspase cleavage of the Z-WEHD substrate was read on a standard plate reader. A caspase-1–specific inhibitor (Ac-YVAD-CHO) was added to parallel samples to confirm specific activity, and wells without cells were run to control for background signal. The values from both the Ac-YVAD-CHO and blank control were subtracted from the Z-WEHD substrate sample value, representing caspase-1 activity.

Immunoblots of cell lysates were probed with the following antibodies: anti-MDA5 (Cell Signaling Technology 3743), anti-LC3 (Cell Signaling Technology 12741), anti–caspase-1 (eBioscience 5B10), or anti–β-actin–HRP (Abcam 49900).

### Microscopy.

Confocal microscopy was used to determine *M*. *tuberculosis*–induced autophagic flux and colocalization of *M*. *tuberculosis* with the autophagosome marker LC3B and the autophagy receptor p62 in J774.1 macrophages. Briefly, the cells were allowed to adhere to sterile glass coverslips placed in 6-well tissue culture plates, prestimulated with IFN-γ, as described above, and infected with an *M*. *tuberculosis* CDC1551 strain ectopically expressing GFP at an MOI of 10 for 6 hours. Cells were fixed, permeabilized, and immunostained using either anti-LC3 antibody (Novus NB100-2220) or anti-p62 antibody (Sigma-Aldrich P0067). Cells were then washed and incubated with Alexa Fluor 647–conjugated secondary antibody (Thermo Fisher Scientific). Hoechst 33342 was used for nuclear staining. Image acquisition was carried out using an LSM700 confocal microscope at ×63 original magnification. Image processing and analysis were done using Fiji (https://github.com/fiji/fiji) and Imaris 9.7 software (Oxford Instruments). For LC3 and p62 quantification, perinuclear puncta were counted in a minimum of 200 cells in different fields using Imaris 9.7 and GraphPad Prism software.

### Infection of mice with M. tuberculosis, assessment of bacterial load, and survival assay.

Female MDA5^–/–^ and WT C57BL/6 mice were infected with the *M*. *tuberculosis* H37Rv strain via the aerosol route using the Glas-Col Inhalation Exposure System with an inoculum that implanted about 2.1 log_10_ CFU in the lungs at day 1 (*n* = 3 WT and *n* = 2 MDA5^–/–^). Both groups of mice for the bacterial burden and time-to-death study were infected in the same chamber in 1 aerosol run. The general appearance and body weight of mice were monitored at least weekly throughout all experiments. All infections, housing of infected mice, and handling of infectious materials were carried out under biosafety level 3 containment in dedicated facilities.

After 4 weeks of infection, 11 mice from each group were sacrificed to evaluate organ bacterial burden. Lungs and spleens were aseptically removed, weighed, placed in 2.5 mL sterile PBS for 24–48 hours at 4°C, examined for gross pathology, and manually homogenized. Homogenates were serially diluted, and 0.5 mL of the homogenate was plated on Middlebrook 7H11 agar (Difco, Thermo Fisher Scientific) supplemented with 10% (vol/vol) OADC, 0.5% (vol/vol) glycerol, 10 mg/mL cycloheximide, 50 mg/mL carbenicillin, 25 mg/mL polymyxin B, and 20 mg/mL trimethoprim (Sigma-Aldrich). Plates were incubated at 37°C for 3–4 weeks. The number of colonies was counted and expressed as log_10_ CFU per organ.

For the mouse time-to-death experiment, 12 mice from each group were infected as described above and monitored for the severity of clinical signs until their death due to TB. Morbid mice were killed according to Institutional Animal Care and Use Committee protocol, and time to death was counted on the day of sacrifice.

### Statistics.

For comparisons between groups, 2-tailed parametric unpaired *t* test, 1-way ANOVA with Tukey’s posttest, or 2-way ANOVA with Tukey’s posttest was used wherever appropriate. Statistical analysis was performed using GraphPad Prism 9 software; *P* less than 0.05 was defined as significant.

### Study approval.

All animal procedures were approved by the Institutional Animal Care and Use Committee of the Johns Hopkins University School of Medicine.

### Data availability.

Values for all data points in graphs are reported in the Supporting Data Values file and are available from the corresponding author upon request.

## Author contributions

CKB and WRB designed the research. CKB, AKS, SK, and SL performed the experiments. PT contributed to autophagy-related experiments. CKB, AKS, SK, GS, and WRB analyzed the data. CKB, GS, and WRB wrote the paper. WRB provided overall supervision of the study. All the authors read, edited, and approved the manuscript.

## Supplementary Material

Supplemental data

Supporting data values

## Version 1 09/19/2023

In-Press Preview

## Version 2 10/23/2023

Electronic publication
