# Supplementary material for: MDA5 RNA-sensing pathway activation by Mycobacterium tuberculosis promotes innate immune subversion and pathogen survival
Source: JCI Insight. 2023 Oct 23;8(20):e166242. doi: 10.1172/jci.insight.166242 (PMC10619499; doi:10.1172/jci.insight.166242)
Supplement: Supplemental data [file jciinsight-8-166242-s127.pdf]

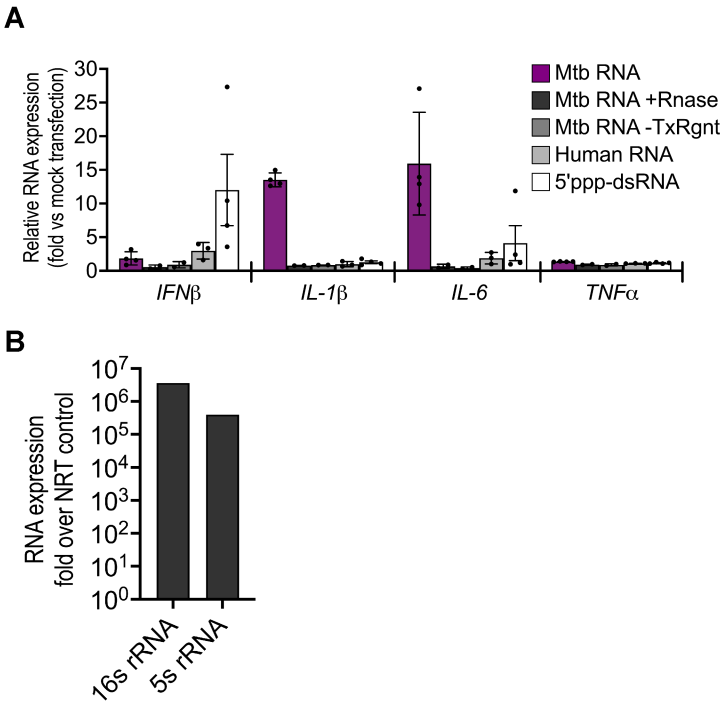

Supplemental Figure 1. *M.tb*-derived RNA induces cytokine response in macrophages.

(A) Resting WT J774.1 macrophages were transfected for 24 h with total *M.tb* RNA, total *M.tb* RNA treated with Rnase V (+Rnase), mock transfection of total *M.tb* RNA (-TxRngt), total human RNA, or the synthetic 5’ triphosphate dsRNA (5’ ppp-dsRNA) ligand. Levels of IFN-β, IL-1β, IL-6 and TNFα RNA were assessed by RT-qPCR. RNA levels were normalized to PolR2A. Data is presented as fold change relative to no transfection control (mean ± s.d., n=4 biological replicates from two independent experiments. **(B)** 5s and 16s rRNA transcripts in total RNA isolated from *M.tb* H37Rv were assessed by qPCR. The qPCR template samples were either cDNA derived from total *M.tb* RNA using reverse transcriptase (RT) or total *M.tb* RNA (NRT). Data is presented as the fold difference between RT and NRT template samples.

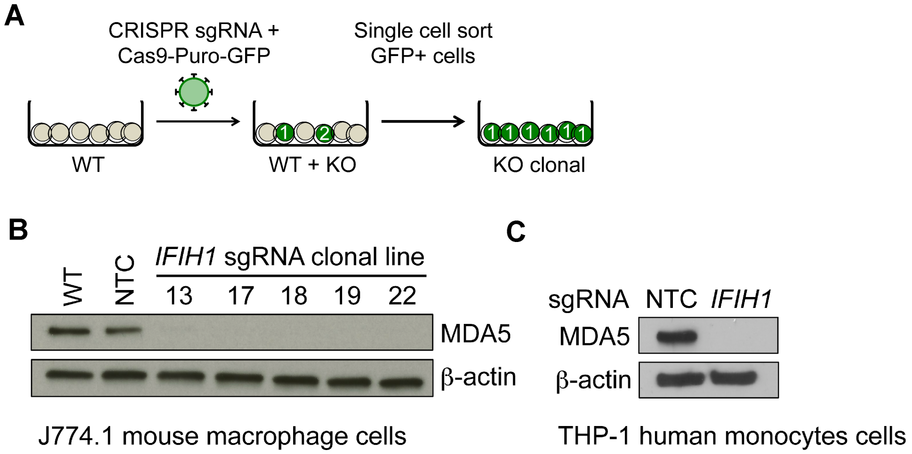

Supplemental Figure 2. Generation and validation of clonal MDA5 knockout (MDA5-KO) macrophage cell lines.

(A) Schematic diagram of the CRISPR-Cas9 lentivirus system used to generate stable and clonal MDA5 knockout J774.1 macrophage cell lines. A CRISPR/Cas9 non-target cell line (NTC) was generated as a control for off-target DNA mutagenesis and cellular passage number. **(B)** MDA5 and b-actin protein levels in IFNy-primed J774.1 macrophages (WT, NTC and clonal MDA5-KO cell lines) detected by immunoblot analysis. **(C)** MDA5 and β-actin protein levels in IFNγ-primed NTC and MDA5-KO THP-1 macrophages (Synthego engineered CRISPR Knockout Cell Clone) detected by immunoblot analysis.

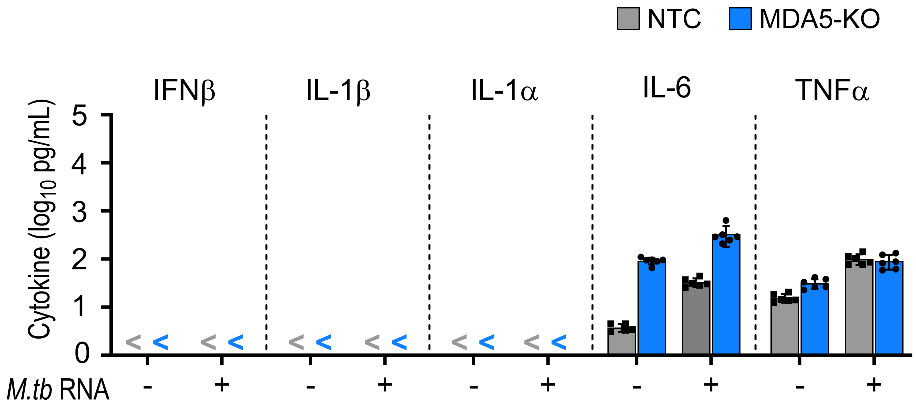

Supplemental Figure 3. *M.tb* RNA stimulation of IL-6 and TNFα production in resting J774.1 macrophages is independent of MDA5.

Resting MDA5-KO or NTC J774.1 cells were transfected with total *M.tb* RNA for 24 h. Levels of IFN-β, IL-1β, IL-6 and TNFα in the culture medium were quantified by multiplex immunoassay (Luminex) (mean ± s.d., n=6 biological replicates from three independent experiments, < = below the limit of quantification).

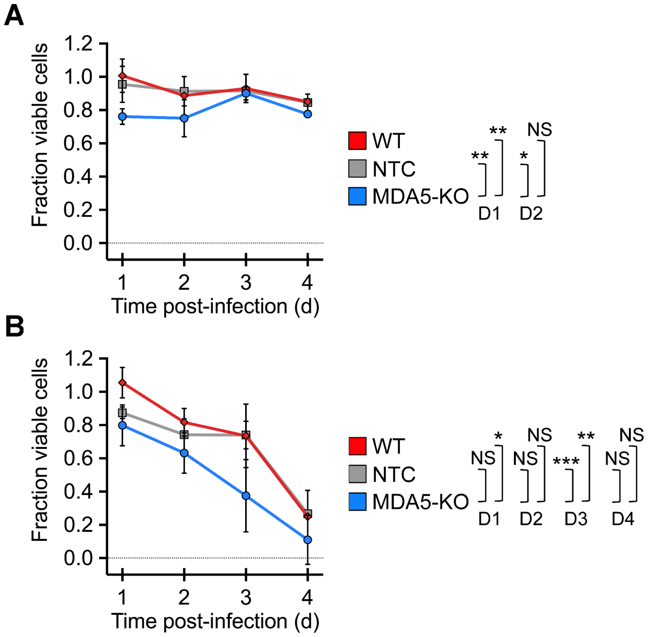

Supplemental Figure 4. Cell viability in J774.1 macrophages infected with *M.tb.*

Cell viability in resting **(A)** or IFNγ-primed **(B)** WT, NTC or MDA5-KO J774.1 cells infected with *M.tb* (MOI of 1:5) quantified by the MTS assay. Data presented as the fraction of colorimetric signal relative to uninfected control cells (mean ± s.d., n=5 from three independent experiments). *P < 0.05, **P < 0.01 and ***P < 0.001 by two-way ANOVA with Tukey's post-test.

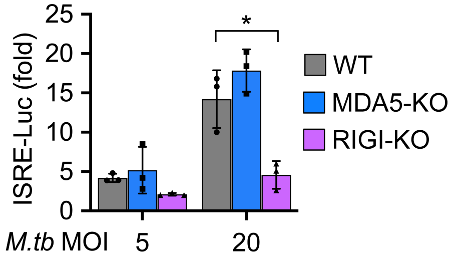

Supplemental Figure 5. Contribution of MDA5 and RIG-I to IRF pathway activation during *M.tb* infection.

RAW-Lucia ISG, RAW-Lucia ISG-MDA5-KO and RAW-Lucia ISG-RIGI-KO cells were infected with the *M.tb* (MOI of 1:5 or 1:20) for 24 h. Levels of IRF-induced Lucia in the cell culture supernatant were measured by the luciferase reporter assay QUANTI-luc. Data presented as fold change relative to no infection control and are the mean ± s.d. from three independent experiments with three biological replicates (n=9). *$P < 0.05$ by two-way ANOVA with Tukey's post-test.

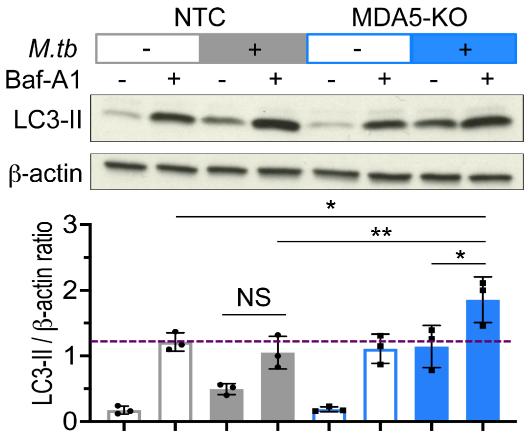

Supplementary Figure 6. Autophagic flux in MDA5-deficient activated macrophages.

LC3-I, LC3-II and β-actin protein levels detected by immunoblot analysis of whole cell lysates from IFNγ-primed MDA5-KO and NTC J774.1 cells infected with *M.tb* (MOI of 1:5) for 21h and then treated with Bafilomycin A1 or vehicle control for 3 h. Densitometric quantification of the LC3-II/β-actin ratio presented as the mean ± s.d. from three independent experiments (n=3). The dotted purple line represents the level of basal autophagic flux in uninfected NTC cells. *$P < 0.05$ and **$P < 0.01$ by two-way ANOVA with Tukey's post-test.

Supplemental Table 1

Mouse Genotype	Day 1 Lung CFU (log)
WT	1.99
WT	2.04
WT	2.23
MDA5 -/-	2.23
MDA5 -/-	2.03

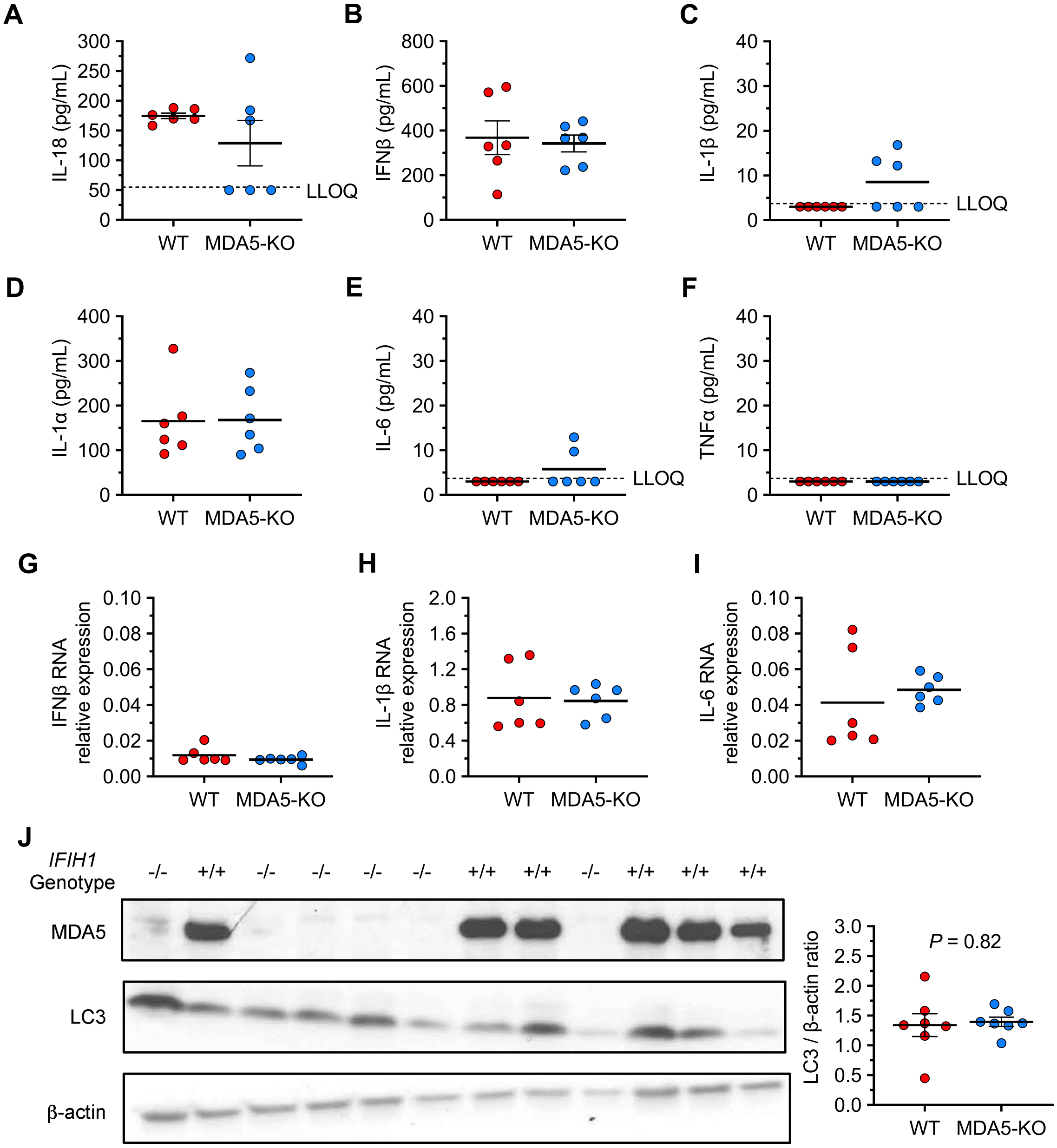

Supplemental Figure 7. Cytokine production and autophagy marker expression in the lungs of WT and MDA5-KO mice infected with *M.tb*.

(A-F) Protein concentration of IL-18 **(A)**, IFNβ **(B)**, IL-1β **(C),** Il-1α **(D)**, IL-6 **(E),** and TNFα **(F)** and in whole lung tissue from MDA5$^{-/-}$ and WT C57BL/6 mice at twelve weeks after aerosol infection were quantified by Luminex multiplex immunoassay (mean ± s.d., n=6 per group). (G-I) RNA levels of IFNβ **(G)** IFNβ **(H)** IL-6 **(I)** in whole lung tissue from MDA5$^{-/-}$ and WT C57BL/6 mice at four weeks after aerosol infection were quantified by RT-qPCR and presented as relative expression of the housekeeping gene PolR2A (mean ± s.d., n=6 per group). **(J)** MDA5, LC3 and β-actin protein levels detected by immunoblot analysis of whole lung lysates from MDA5$^{-/-}$ and WT C57BL/6 mice at twelve weeks after aerosol infection. Densitometric quantification of the LC3/β-actin ratio presented as the mean ± s.d (n=6 per group).

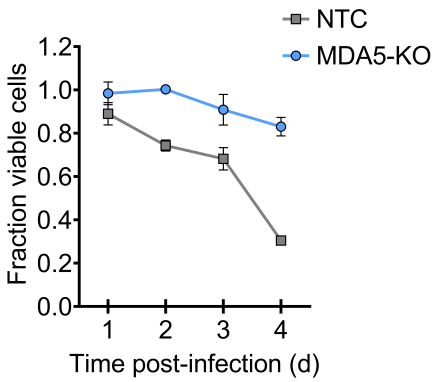

Supplemental Figure 8. Cell viability in Thp-1 macrophages infected with *M.tb.*

Cell viability in PMA-differentiated and IFNγ-primed THP-1 cells (NTC and MDA5-KO) infected with *M.tb* (MOI of 1:5) quantified by the Cell Titer Glo assay. Data presented as the fraction of RLU relative to uninfected control cells (mean ± s.d., n=6 biological replicates from one independent experiment).
